# Persistent Chronic Cough Revealing Late Pulmonary Metastasis of a Solitary Fibrous Tumor More Than A Decade After Resection

**DOI:** 10.7759/cureus.108223

**Published:** 2026-05-04

**Authors:** Esther Y Liu, Geoffrey Modest

**Affiliations:** 1 Medicine, Beth Israel Deaconess Medical Center, Harvard Medical School, Boston, USA; 2 Family Medicine, Beth Israel Deaconess Medical Center, Harvard Medical School, Boston, USA

**Keywords:** chronic cough, metastatic tumor, primary care medicine, rare case report, solitary fibrous tumor (sft)

## Abstract

Persistent chronic cough is a common outpatient symptom, typically caused by gastroesophageal reflux, asthma, or upper airway cough syndrome. Rare causes, including metastatic tumors, may present with nonspecific respiratory symptoms, particularly in patients with a history of malignancy.

A 59-year-old man with a prior left frontal grade II solitary fibrous tumor (SFT) resected in 2012 presented with a 2-3-month history of dry cough. Initial empiric therapy for gastroesophageal reflux and post-nasal drip failed. Chest radiograph revealed multiple bilateral pulmonary nodules, and CT chest showed an 8 × 4.5 cm subcarinal mass. Bronchoscopy with transbronchial needle aspiration confirmed metastatic pulmonary SFT. The patient is now being followed by oncology for staging and treatment planning.

Persistent cough that does not improve with empiric therapy warrants early imaging, especially in patients with prior malignancy. This case highlights that SFT can metastasize many years after initial treatment, presenting with common symptoms such as cough. Timely reassessment can prevent delays in diagnosis and management.

## Introduction

Chronic cough, defined as a cough lasting longer than eight weeks, is a common outpatient complaint and accounts for a substantial proportion of primary care and pulmonary visits. The majority of cases are attributed to gastroesophageal reflux disease, asthma, or upper airway cough syndrome (UACS) and are often managed empirically based on guideline-directed algorithms [1]. However, persistent symptoms despite appropriate therapy should prompt evaluation for less common etiologies, including malignancy.

Solitary fibrous tumors (SFTs) are rare mesenchymal neoplasms that most commonly arise from the pleura but can occur in a variety of extrapleural sites [2]. These tumors were historically referred to as hemangiopericytomas, but this terminology has since been revised based on advances in pathological classification. Although often indolent, SFTs exhibit unpredictable clinical behavior, with risk of local recurrence or distant metastasis even decades after initial resection. Delayed pulmonary metastasis from extrapleural SFTs is particularly rare and may present with nonspecific respiratory symptoms, contributing to delays in diagnosis [3].

Here, we report a case of late pulmonary metastasis of SFT presenting as persistent chronic cough more than 10 years after initial tumor resection, highlighting the importance of maintaining a broad differential diagnosis in patients with refractory symptoms.

## Case presentation

A 59-year-old man with a history of left frontal grade II SFT resected in 2012 with adjuvant radiation and subsequent seizures presented with a 2-3-month history of a dry, persistent cough. He reported that the cough occurred with meals and denied sputum production, hemoptysis, chest pain, dyspnea, fevers, or weight loss. He had no smoking history, no family history of malignancy, and was taking lamotrigine and levetiracetam. Initial lung examination was unremarkable.

Given the suspected temporal association with meals, gastroesophageal reflux disease (GERD) was initially considered, and he was started on a two-month trial of omeprazole. Basic laboratory evaluation, including a complete blood count with differential and comprehensive metabolic panel, was unremarkable.

At follow-up, symptoms persisted without improvement. His wife subsequently noted that the cough was not consistently related to meals and occurred randomly throughout the day. Repeat examination demonstrated new bilateral rhonchi, and post-nasal drip syndrome was considered. Antihistamine therapy was initiated; however, symptoms again failed to improve.

**Figure 1:**
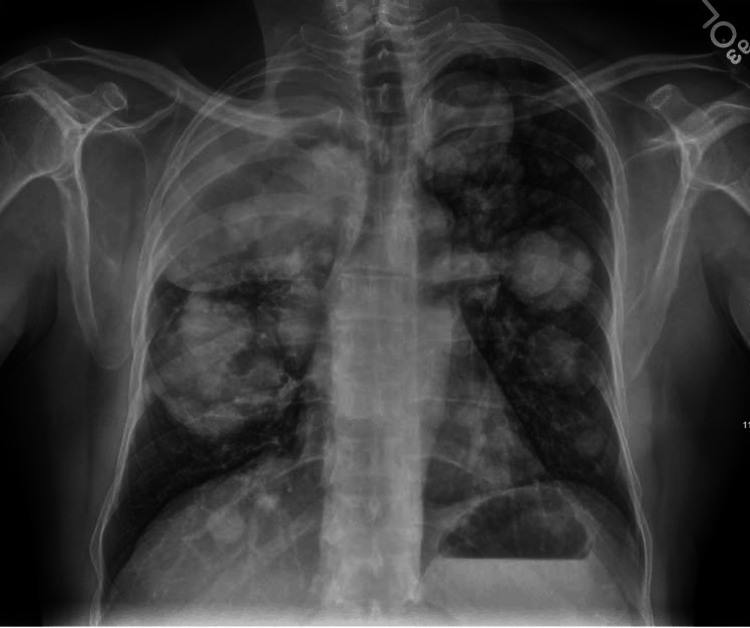
Chest radiograph demonstrating multiple bilateral pulmonary nodules in a patient with a prior solitary fibrous tumor

**Figure 2:**
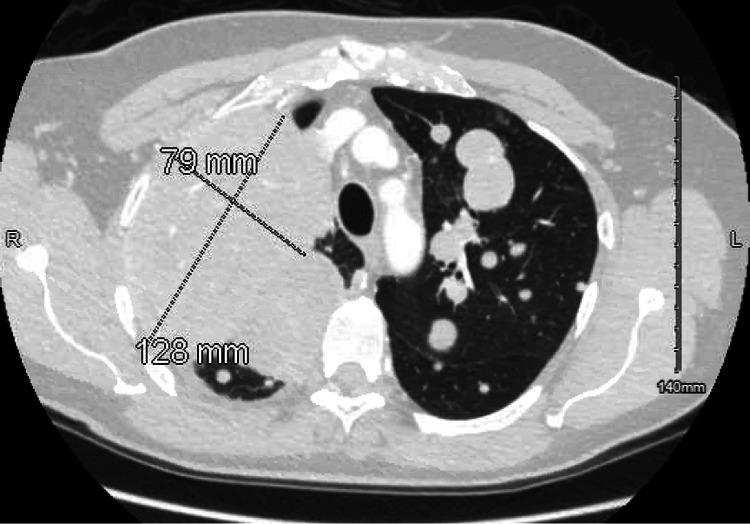
CT scan of the chest showing an 8 × 4.5 cm subcarinal mass concerning for metastasis of the solitary fibrous tumor

Given the persistent cough despite empiric therapy, imaging was obtained. Chest radiograph demonstrated multiple bilateral pulmonary nodules and masses (Figure 1). Subsequent computed tomography (CT) of the chest revealed innumerable bilateral pulmonary nodules of varying sizes along with an 8 × 4.5 cm subcarinal mass concerning for metastatic disease (Figure 2).

The patient underwent bronchoscopy with transbronchial needle aspiration of pulmonary nodules and mediastinal lymph nodes. Histopathologic evaluation of the lymph node demonstrated metastatic hemangiopericytoma, consistent with metastatic solitary fibrous tumor in the clinical context of the patient’s prior primary tumor. The patient was diagnosed with metastatic SFT involving the lungs and mediastinal lymph nodes. He has since established care with oncology and is undergoing further staging with plans for systemic therapy. The oncology team will guide treatment response and long-term management.

## Discussion

Chronic cough is a common presenting symptom in outpatient practice. While GERD, asthma, and UACS account for most cases, clinical guidelines emphasize that a persistent cough after appropriate empiric therapy warrants reassessment and chest imaging rather than continued management of benign causes [4]. In typical cases, GERD-associated chronic cough has been shown to improve within four to eight weeks of PPI therapy [5]. Other common causes, such as asthma or UACS, are often expected to respond within days to weeks of appropriate therapy [6]. Failure to improve within these expected timeframes should prompt reconsideration of the diagnosis and further evaluation.

Patients with a history of malignancy represent an important exception to empiric treatment algorithms. In these patients, clinicians should maintain a lower threshold for early chest imaging when evaluating persistent cough rather than pursuing prolonged empiric therapy. Late recurrence of solitary fibrous tumors has been documented, with reported recurrence rates of approximately 10-30% and metastatic risk as high as 35-45% in some series. Notably, recurrences may occur more than a decade after initial treatment, and can present with nonspecific symptoms, such as cough, increasing the risk of delayed recognition [7].

Formerly termed hemangiopericytomas, SFTs are rare mesenchymal neoplasms with highly variable behavior [8]. Although many are histologically benign, they may recur or metastasize years after initial treatment, and pulmonary involvement is very rare. The most commonly reported metastatic sites of SFT include the lungs, liver, and bone [9,10]. Dissemination typically occurs via hematogenous spread, while lymph node metastasis is relatively uncommon and only rarely described, as seen in this patient [11]. Distant pulmonary metastases of SFT have been reported even a decade after primary tumor resection, highlighting the unpredictable and potentially long latent course of these neoplasms [8]. This prolonged latency underscores the importance of long-term surveillance in affected patients and maintaining a high index of suspicion for metastatic disease when new lesions arise, even after extended disease-free intervals.

This case highlights the importance of reassessing a persistent cough when the clinical trajectory does not align with expected improvement. Importantly, patients with any malignant history require earlier imaging, as even remote cancers can recur or metastasize years later. Specifically, early chest radiography represents a low-cost, low-risk initial diagnostic step and should be considered early in the evaluation of persistent cough. Late recurrence of rare tumors, such as SFT, can manifest with common symptoms, such as cough, and may be easily overlooked if clinicians anchor on benign explanations.

## Conclusions

In summary, persistent chronic cough requires careful reassessment when symptoms do not improve after treatment for common etiologies. Patients with any history of malignancy should undergo earlier chest imaging, as late recurrence or metastasis can present with nonspecific symptoms such as cough. This case illustrates how timely re-evaluation prevents delays in identifying rare but clinically significant causes of cough such as metastatic SFT.

## References

[1] Alhajjaj MS, Sankari A, Bajaj P (2025). Chronic cough. StatPearls [Internet].

[2] Boualoy T, Collier SA, Abodunrin FO, Menon G (2025). Solitary fibrous tumors. StatPearls [Internet].

[3] Matsuishi K, Eto K, Morito A (2021). Retroperitoneal fibrous tumor recurring as lung metastases after 10 years: a case report. Surg Case Rep.

[4] Hu X, Zhang K, Liu T, Zhu XJ (2025). Chronic cough: a review and prospects. Medicine (Baltimore).

[5] Park HJ, Park YM, Kim JH, Lee HS, Kim HJ, Ahn CM, Byun MK (2017). Effectiveness of proton pump inhibitor in unexplained chronic cough. PLoS One.

[6] Pratter MR (2006). Chronic upper airway cough syndrome secondary to rhinosinus diseases (previously referred to as postnasal drip syndrome): ACCP evidence-based clinical practice guidelines. Chest.

[7] Georgiesh T, Aggerholm-Pedersen N, Schöffski P (2022). Validation of a novel risk score to predict early and late recurrence in solitary fibrous tumour. Br J Cancer.

[8] Afreen S, Wu X, Miranda ME, Lazarevic M (2024). A rare case of solitary fibrous tumor of the lung parenchyma: case report. J Surg Case Rep.

[9] Ota H, Kawai H (2011). A case of solitary fibrous tumor of the pleura with pulmonary metastasis 21 years after initial surgery. Haigan.

[10] Ratneswaren T, Hogg FR, Gallagher MJ, Ashkan K (2018). Surveillance for metastatic hemangiopericytoma-solitary fibrous tumors-systematic literature review on incidence, predictors and diagnosis of extra-cranial disease. J Neurooncol.

[11] Mearini E, Cochetti G, Barillaro F, Fatigoni S, Roila F (2014). Renal malignant solitary fibrous tumor with single lymph node involvement: report of unusual metastasis and review of the literature. Onco Targets Ther.

